# The complete mitochondrial genome of *Histiostoma blomquisti* (Acari: Histiostomatidae)

**DOI:** 10.1080/23802359.2016.1219633

**Published:** 2016-09-03

**Authors:** Chih-Chi Lee, John Wang

**Affiliations:** Biodiversity Research Center, Academia Sinica, Taipei, Taiwan

**Keywords:** Histiostomatidae, mitochondrial genome, mites, red imported fire ants

## Abstract

The mite *Histiostoma blomquisti* is a microorganism feeder that uses the red imported fire ant (*Solenopsis invicta*) as a phoretic carrier for dispersal. We sequenced the *H. blomquisti* mitogenome using next-generation sequencing methods. The circular mitogenome of *H. blomquisti* is 15,892 bp and is composed of 13 protein-coding genes, two ribosomal RNA genes, 22 transfer RNAs, and 6 non-coding regions >100 bp. Most tRNAs are highly reduced, like those found in other Acariformes. Phylogenetic analysis based on the concatenated nucleotide sequence of the 13 protein-coding genes supports Histiostomatid mites forming the basal-most lineage in Astigmata.

Histiostomatid mites are filter feeders whose phoretic second stage nymphs, or deutonymphs, disperse by attaching to arthropods. The deutonymphs of *Histiostoma blomquisti* can often be found on the cuticle of red imported fire ant (*Solenopsis invicta*) queens (Sokolov et al. 2003; Wirth & Moser 2010). Here, we present the first complete Histiostomatid mitogenome.

For sample collection, we scraped approximately 330 deutonymphs from the abdominal cuticle of *S. invicta* queens. Queens were from colonies of the polygyne social form collected from Taoyuan City, Taiwan (24**°**56′06.71″N, 121**°**12′40.39″E). We mounted three mites onto slides (ASIZ01000010 - ASIZ01000012) and deposited them at the Biodiversity Research Museum, Academia Sinica, Taiwan.

To obtain enough DNA for sequencing, we extracted genomic DNA with the QIAamp^®^ DNA Micro Kit (QIAGEN) and then amplified the DNA with the GenomiPhi^TM^ V3 DNA Amplification Kit (GE Healthcare Life Sciences). We sequenced the amplified DNA on the Illumina MiSeq platform (average library insert size 579 bp; paired-end read length 301 bp).

For assembly and annotation of the mitogenome, we preprocessed raw sequence reads (cutadapt v1.9.1 (Martin 2011); parameters: -a -q 20 -m 70) and conducted *de novo* whole genome assembly (IDBA-UD (Peng et al. 2012); parameters: –mink 40 –min_count 4 –min_support 2). We identified two mitogenome contigs from the genome assembly. After, we conducted PCR, cloning, and sequencing to bridge the two contigs into a circular mitogenome. We annotated protein coding genes (PCGs) using MITOS (Bernt et al. 2013) and OrfFinder (Sayers et al. 2011). We annotated tRNAs using ARWEN (Laslett & Canbäck 2008), tRNAscan-SE (Lowe & Eddy 1997), and manual identification based on the anticodon and predicted secondary structure.

The complete mitogenome of *H. blomquisti* (GenBank: KX452726) is 15,892 bp, which is the largest Sarcoptiformes mitogenome to date. The nucleotide composition is AT biased (70%), similar to other Acariformes. The *H. blomquisti* mitogenome contains 13 PCGs, 2 rRNAs, and 22 tRNAs, typical for most animals. However, the tRNAs range in size from 45–59 bp, which are shorter than usual animal tRNAs (circa 75–85 bp). The smallest four tRNAs (45–50 bp, *trnR*, *trnA*, *trnV*, and *trnS2^(UCN)^*) could only be annotated manually. Among the 13 PCGs, 12 use ATN as the start codon (N, any nucleotide) while *nad3* uses TTG. The stop codons of five PCGs are incomplete (T- for *nad2* and *CYTB*; TA- for *cox2*, *nad1*, and *nad6*). The two largest non-coding regions (903 and 413 bp) are separated by a tRNA. As the former is adjacent to rrnS, we presume it corresponds to the control region.

We inferred the phylogenetic relationship of 15 mites within Acariformes, including at least one representative from each family, using the concatenated nucleotide sequences of the 13 PCGs that were aligned based on the corresponding amino acid translation (Edgar 2004). Both maximum likelihood (GTRGAMMA model, RAxMLGUI (Silvestro & Michalak 2012)) and Bayesian inference (GTR + I + Γ model, MrBayes 3.2.5 (Ronquist et al. 2012)) yielded the same phylogeny indicating that Histiostomatid mites form the basal-most sequenced lineage within Astigmata (Figure 1).

**Figure 1.**
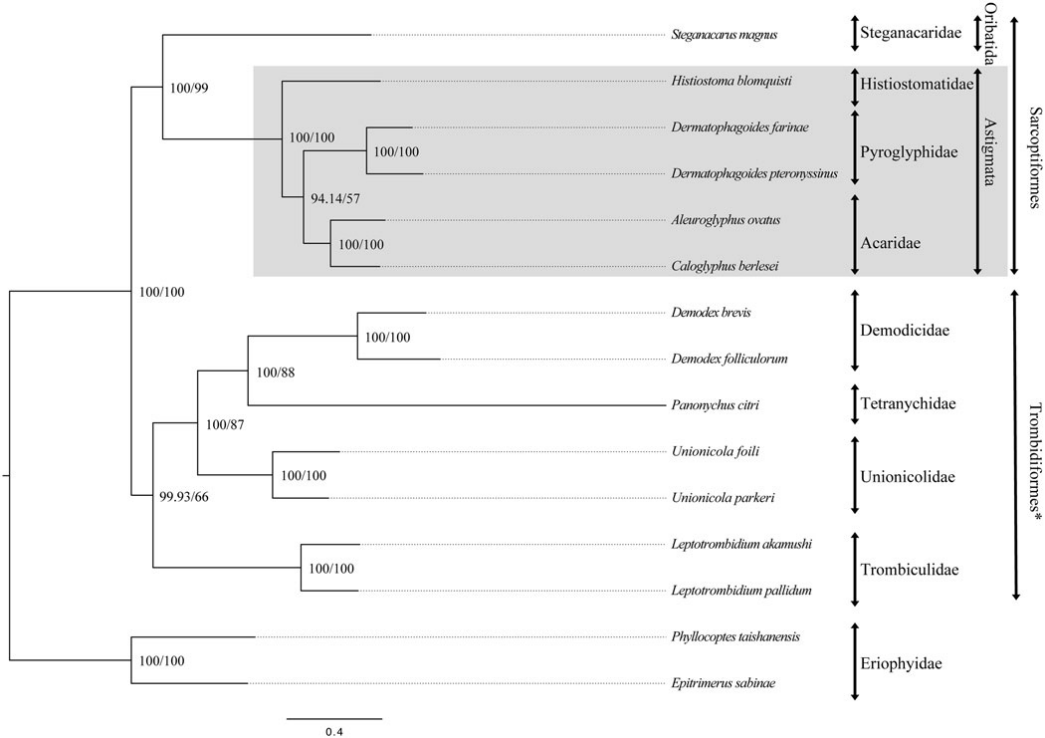
Molecular phylogeny of *Histiostoma blomquisti* and 14 other Acariformes based on the concatenated nucleotide sequences of 13 PCGs. The phylogenetic tree was constructed by the Bayesian inference and maximum-likelihood methods under GTR + I + Γ and GTRGAMMA models, respectively. The numbers at each node indicate the posterior probability (100,000 generations, sampled every 100 generations) and the bootstrap probability (1000 replicates) resulting from the analyses. The mitogenome accession numbers for tree construction are listed as follows: *Aleuroglyphus ovatus* (KC700022), *Caloglyphus berlesei* (KF499016), *Dermatophagoides pteronyssinus* (EU884425), *D. farinae* (NC_013184), *Steganacarus magnus* (EU935607), *Histiostoma blomquisti* (this study: KX452726), *Demodex brevis* (KM114225), *Demodex folliculorum* (KM114226), *Phyllocoptes taishanensis* (KR604967), *Epitrimerus sabinae* (KR604966), *Panonychus citri* (HM189212), *Leptotrombidium pallidum* (AB180098), *L. akamushi* (AB194045), *Unionicola foili* (EU856396), and *U. parkeri* (HQ386015). Astigmata (shaded box). *We rooted the phylogenetic tree using Eriophyidae based on their exclusion from Trombidiformes in Xue et al. (2016).
